# Valorization of outer tunic of the marine filter feeder *Ciona intestinalis* towards the production of second-generation biofuel and prebiotic oligosaccharides

**DOI:** 10.1186/s13068-021-01875-4

**Published:** 2021-01-28

**Authors:** Kateřina Hrůzová, Leonidas Matsakas, Anthi Karnaouri, Fredrik Norén, Ulrika Rova, Paul Christakopoulos

**Affiliations:** 1 Biochemical Process Engineering, Division of Chemical Engineering, Department of Civil, Environmental and Natural Resources Engineering, Luleå University of Technology, 971 87 Luleå, Sweden; 2 N-Research AB, Gränsgatan 17, 453 30 Lysekil, Sweden

**Keywords:** *Ciona intestinalis*, Tunicate, Bioethanol, Cellobiose, Prebiotics

## Abstract

**Background:**

One of the sustainable development goals focuses on the biomass-based production as a replacement for fossil-based commodities. A novel feedstock with vast potentials is tunicate biomass, which can be pretreated and fermented in a similar way to lignocellulose. *Ciona intestinalis* is a marine filter feeder that is cultivated to produce fish feed. While the inner tissue body is used for feed production, the surrounding tunic remains as a cellulose-rich by-product, which can be further separated into outer and inner tunic. Ethanol production from organosolv-pretreated whole-tunic biomass was recently validated. The aim of the present study was to evaluate the potential of organosolv pretreated outer-tunic biomass for the production of biofuels and cellobiose that is a disaccharide with prebiotic potential.

**Results:**

As a result, 41.4 g/L of ethanol by *Saccharomyces cerevisiae*, corresponding to a 90.2% theoretical yield, was achieved under the optimal conditions when the tunicate biomass was pretreated at 195 °C for 60 min at a liquid-to-solid ratio of 50. In addition, cellobiose production by enzymatic hydrolysis of the pretreated tunicate biomass was demonstrated with a maximum conversion yield of 49.7 wt. %.

**Conclusions:**

The utilisation of tunicate biomass offers an eco-friendly and sustainable alternative for value-added biofuels and chemicals. The cultivation of tunicate biomass in shallow coastal sea improves the quality of the water and ensures sustainable production of fish feed. Moreover, there is no competition for arable land, which leaves the latter available for food and feed production.

## Background

The industrial production of biomass-derived value-added commodity products instead of petrochemicals is gaining increasing interest from an environmental and economical point of view [1]. The increasing demand for alternatives to fossil resources for the production of fuels, renewable materials, polymers, food supplements and pharmaceuticals [2], as well as the sustainable development goals set by various organizations and governments worldwide have promoted research on biomass as a platform for the production of chemicals [3]. Regarding the biofuels, all three generations of biofuels have considerable advantages and disadvantages. First-generation biofuels have been produced from edible biomass, such as starch, sugar, animal fat, and vegetable oils. Despite the mature technology and good results, the production of biofuels competes with food and feed production. Production of second-generation biofuels is steadily moving from pilot scale to commercial scale [4–6]. Even though these biofuels are generated from non-edible biomass, including waste vegetable oils, municipal waste, and lignocellulosic materials, they still put a burden on arable land that could be used by the food and feed industry or for forestry [7]. Third-generation biofuels have been produced by algae via photosynthesis and, owing to their novelty, application of the technology at a commercial scale needs substantial improvement and optimisation [8]. In our previous study [9], the tunicate biomass validated as a new potential feedstock for second-generation biofuel production with the considerable advantage compare to standard second-generation biofuels which is the independence on the arable land. *Ciona intestinalis* is a marine filter feeder tunicate, whose body consists of two main parts: the inner body tissue and the surrounding tunic [10]. These tunicates can easily be cultivated in large quantities and harvested at 10 tonnes per day. Currently, the main product is the inner body tissue, which is processed into fish feed, while the tunic by-product is used for biogas production and as fertilizer [11]. The great side effect of the tunicate biomass production is a reduction of the water eutrophication as the tunicates filter the excess carbon and nitrogen from the water [12]. Thus, the sustainability is getting more pronounced in all the part of the production cycle. Tunicates are the only invertebrate organism group capable of producing cellulose, which is present in the tunic part at up to 17.1 wt. % of dry biomass [9, 13]. The main difference between tunicate cellulose and lignocellulose is the composition of the surrounding matrix. Whereas lignocellulosic cellulose is bound by lignin and hemicellulose, tunicate cellulose is bound by proteins and polysaccharides [13]. Despite the difference in biomass composition, good results were obtained when organosolv pretreatment was applied for the fractionation of whole-tunicate biomass [9]. To further improve bioethanol yields using tunicate biomass, the tunic can be separated in an outer and inner (mantle) part, of which the former has a higher cellulose content. Compared to other marine biomass sources, the results obtained with pretreated tunicate biomass are 4- to 10-folds higher. The study with aquatic plant *Eichhornia crassipes* has resulted in the ethanol concentration of 9.6 g/L [14]. Similar results were reached with different types of algae *Laminaria japonica*, *Gracilaria* sp., and *Saccharina japonica* where the ethanol concentration was between 4.9 and 10.9 g/L [15–17].

As a lignin-free cellulose-rich material, outer tunic can also serve as a starting material for the isolation of cello-oligosaccharides (COS), apart from the production of bioethanol. COS belong to a group of functional oligosaccharides called *prebiotics*, which includes compounds that are either entirely non-digestible or slowly accumulated by the gastrointestinal tract and thus, they remain available as carbon source for the beneficial bacteria that consist the microbiota of the animal and human colon [18]. COS, as well as other biomass-derived carbohydrates such as xylo-oligosaccharides, which can be produced from the hemicellulosic part of agricultural and forestry biomass, consist of β-glycosidic bonds, therefore they are resistant to digestion by gastrointestinal enzymes. In our previous studies, we have verified the ability of COS production (comprised mainly of cellobiose) from non-edible lignocellulosic biomass and we have proved that these oligosaccharides are able to support the growth of some probiotic strains belonging to *Lactobacilli* species [19]. COS can be produced by acid hydrolysis of cellulose; however, employing a strategy of controlled enzymatic hydrolysis is considered more attractive due to the milder reaction conditions and easy recovery of final products. Cellulases with processive mode of action, more specifically cellobiohydrolases belonging to glycoside hydrolase family 7 (CBH7) and endoglucanases belonging to glycoside hydrolase family 5 (EG5), have been proved out to be the key enzymes for the production of COS from cellulose-rich substrates [20].

**Fig. 1:**
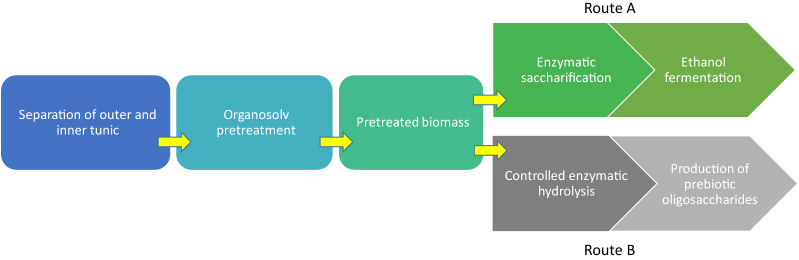
Flow diagram of the novel processes suggested for the valorisation of outer tunic. The valorisation flow diagram of outer tunic fraction towards bioethanol (*Route A*) and cellulose-derived oligosaccharides with prebiotic potential (*Route B*)

The aim of the present study was to evaluate the valorization potential of the outer tunic fraction towards the production of bioethanol and prebiotic oligosaccharides. The effect of the additional separation step of the inner part of tunic on the ethanol yields obtained from tunicate biomass was studied. Organosolv pretreatment was applied to outer tunic biomass, followed by enzymatic saccharification and fermentation (Fig. 1, *Route A*). The results were compared to those obtained using whole-tunic biomass. Moreover, for the first time, outer tunic was examined as a feedstock to produce cello-oligosaccharides as potential food-grade prebiotics (Fig. 1, *Route B*).

## Results and discussion

### Composition of the pretreated biomass

The potential of whole-tunicate biomass as a feedstock for second-generation bioethanol production has been proven recently [9]. In the present study, only the outer tunic, which has higher cellulose content than whole tunic, was used as raw material for fractionation by organosolv pretreatment. Although the inner part of the tunic is not used for ethanol production in this scenario, it can still be used for biogas and fertilizer production [12]. Therefore, all of the by-products are utilized to provide sustainable processes. The composition of the untreated outer tunic was 28.8 wt.% carbohydrates (22.6% glucan and 5.2% galactan), 28.9 wt.% protein, 2.3 wt.% lipid, and 21.8 wt.% ash (Table 1). In comparison, the untreated whole-tunic composition was 21.6 wt.% carbohydrates (including 17.1% glucan, 2.3% mannan and 2.2% galactan), 26.2 wt.% proteins, 7.7 wt.% lipids, and 24.2 wt.% ash [9].

**Table 1:** Chemical composition of untreated and pretreated outer tunic biomass

Biomass sample	Biomass solubilisation (%)	Cellulose		Protein		Ash		Lipid		Mass closure (%)
		wt. (%)	solub. (%)	wt. (%)	solub. (%)	wt. (%)	solub. (%)	wt. (%)	solub. (%)	
Untreated outer tunic	0.0	22.6 ± 1.0	0.0	28.9 ± 2.9	0.0	21.8 ± 0.4	0.0	2.3 ± 0.0	0.0	75.6
Pretreated (LSR 10)	55.5	45.0 ± 0.1	11.4	21.0 ± 2.1	27.3	19.6 ± 0.1	60.0	3.7 ± 0.1	28.4	89.3
Pretreated (LSR 25)	59.1	44.7 ± 0.2	19.1	14.8 ± 1.5	48.8	17.8 ± 0.0	66.6	4.6 ± 0.2	18.2	81.9
Pretreated (LSR 50)	59.1	53.9 ± 0.3	2.5	10.5 ± 1.1	63.7	15.3 ± 0.0	71.3	2.5 ± 0.0	55.5	82.2
Pretreated (LSR 75)	67.1	65.0 ± 0.0	5.4	7.1 ± 0.7	75.4	12.6 ± 0.5	81.0	1.5 ± 0.1	78.5	86.2

First, the effect of different temperatures was examined (data not shown) and a cooking temperature of 195 °C was selected. Since addition of catalyst during organosolv pretreatment of whole tunic had failed to improve the composition of pretreated biomass or the yields of enzymatic saccharification and fermentation [9], acid or base catalysts were not included in this study. In contrast, adjusting the liquid-to-solid ratio (LSR) had been shown to exert a significant beneficial effect on ethanol production from whole tunic [9] and, hence, LSR was included as a variable in organosolv pretreatment of the outer tunic.

Based on the above selection criteria, four different samples, each with a different LSR, were obtained. The cellulose content increased significantly after organosolv pretreatment compared to the initial untreated biomass (22.6 wt. %), reaching a final content between 44.7 wt. % (LSR 25) and 65.0 wt. % (LSR 75). Generally, a higher LSR during pretreatment led to higher solubilisation of protein, ash, and lipids. The exception to this rule was LSR 25, whereby cellulose exhibited the highest (19.1%) and lipids the lowest (18.2%) solubilisation. At the highest LSR (75), 65.0% of pretreated biomass was cellulose, while the solubilisation of protein, ash, and lipids reached 75.4, 81.0, and 78.5%, respectively. These results are comparable to those obtained in our previous study [9], whereby the cellulose content at LSR 10, 25, 50, and 75 was 48.5 wt.%, 47.4 wt.%, 56.3 wt.% and 58.5 wt.%, respectively. Compared to the outer tunic only, overall protein (80.5–88.7%) and lipids (83.1–95.8%) solubilisation was slightly higher when whole tunicate biomass was used; whereas ash solubilisation was slightly lower (54.4–75.4%) and final ash content in pretreated whole biomass was roughly two times higher (20.1–32.3% w/w). An elevated ash content may hamper the overall saccharification process and can influence the fermentation performance [21]. Hence, summarizing all the above results, organosolv pretreatment of outer tunic biomass that was retrieved after the additional separation step, resulted in a slightly more efficient fractionation compared to whole-tunic biomass and yielded a cellulose-rich solid pulp.

### Enzymatic saccharification potential

**Fig. 2:**
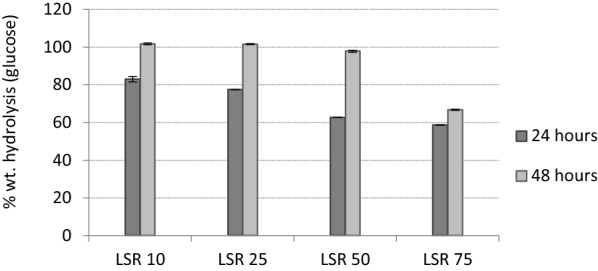
% wt. conversion of outer tunic to glucose by Cellic CTec2

Trials with low solids (3 wt.% dry solids) were performed to assess the potential of the pretreated outer tunic for enzymatic saccharification (Fig. 2). The sample corresponding to LSR 10, 25 and 50 resulted in complete saccharification of cellulose and thus in very high glucose concentration (15.3, 15.1, and 17.6 g/L, respectively). However, a further increase in LSR to 75 caused a drop in the glucose concentration to similar levels as LSR 10 and 25 (14.5 g/L) and considering the higher cellulose content it resulted to a reduced saccharification yield of 66.8%.

Based on these values, the saccharification performance of outer tunic appeared higher than in the case of whole tunic, whereby LSRs of 10, 25, 50, and 75 resulted in glucose concentrations of 11.4, 10.5, 13.0, and 9.2 g/L, respectively, while conversion yields ranged from 47.4% (LSR 75) to 70.3% (LSR 10) [9].

### Synergistic interaction of Cellic CTec2 with cellobiohydrolase CBHI

**Fig. 3:**
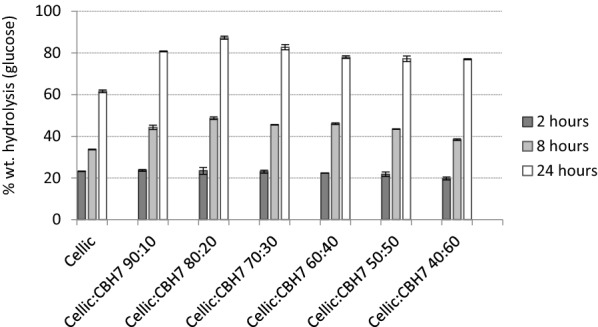
% wt. conversion of outer tunic to glucose by different combinations of Cellic CTec2 and CBHI. The total enzyme load was kept constant through the evaluation of the different combinations of Cellic CTec2 and CBHI

To test whether it would be possible to increase the hydrolysability of the outer tunic (LRS 75) while maintaining the total enzyme loading, Cellic CTec2 was replaced by cellobiohydrolase CBHI in different percentages. The ratios of Cellic CTec2 to cellobiohydrolase were 90:10, 80:20, 70:30, 60:40, 50:50, and 40:60. CBHI was selected due to the fact that this enzyme constitutes the main activity of most fungal cellulolytic systems [22] and most of the cellulolytic cocktails that are currently commercially available. Our results showed that synergistic effects between CBHI and Cellic CTec2 are observed in all different combinations, as depicted in Fig. 3. Even when 10% of the cellulolytic mixture is replaced by CBHI, glucose yield increases from 61.6% to 80.7 wt. %. The increase in glucose yield is more profound when CBHI is added at 20% of the total enzyme loading, resulting in 87.3 wt. % hydrolysis.

### Ethanol fermentation

**Fig. 4:**
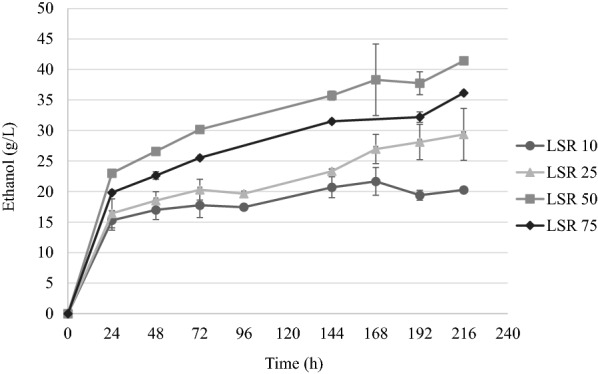
Time-course of ethanol production (g/L) using outer tunic biomass at 15 wt. % dry biomass

Tunicate biomass is a challenging material due to its hygroscopic properties and the tendency to absorb all the available water. When biomass content exceeds 10 wt. % dry solids in a reaction, there is practically no free water in the cultivation flask and the saccharification process does not lead to any visible change in the consistency of the slurry. However, a 15 wt. % dry solids biomass content has been recently shown to be effectively hydrolyzed [9]; hence, this condition was also applied here for the fermentation of pretreated outer tunic biomass (Fig. 4).

High ethanol concentrations and yields were obtained already from the first day of fermentation. After 24 h of fermentation, the ethanol yields reached between 35.8 and 50.1% and the ethanol concentration were between 15.3 and 23.0 g/L (Table 2). The fermentation of pretreated outer tunic at LSR 10 resulted in 21.7 g/L ethanol, corresponding to a yield of 56.6%. Augmenting the LSR to 25 increased ethanol concentration to 29.4 g/L and yield to 77.2%. The highest ethanol concentration (41.4 g/L), amounting to a yield of 90.2%, was reached with biomass pretreated at LSR 50. As before, a further increase in LSR to 75 did not improve further the concentration (36.2 g/L) or yield (65.4%).

**Table 2:** Ethanol concentration and yields obtained after fermentation at 15% w/w dry mass content

Biomass sample	Cellulose %	Ethanol at 24 h		Highest ethanol concentration		
		Ethanol (g/L)	Yield (%)	Ethanol (g/L)	Time (h)	Yield (%)
Pretreated (LSR 10)	45.0	15.3 ± 1.6	39.9 ± 4.2	21.7 ± 2.3	168	56.6 ± 5.9
Pretreated (LSR 25)	44.7	16.4 ± 2.4	43.1 ± 6.2	29.4 ± 4.3	216	77.2 ± 11.2
Pretreated (LSR 50)	53.9	23.0 ± 0.0	50.1 ± 0.0	41.4 ± 0.3	216	90.2 ± 0.7
Pretreated (LSR 75)	65.0	19.8 ± 0.2	35.8 ± 0.3	36.2 ± 0.1	216	65.4 ± 0.2

Generally, the results obtained with outer tunic biomass were slightly lower in terms of ethanol concentration and yields compared to those obtained with whole tunic. For example, ethanol concentration and yield with whole-tunic biomass were 34.9 g/L and 84.5% for LSR 10, and 38.7 g/L and 78.3% for LSR 75, respectively. In absolute terms, though, the highest concentration was obtained with outer tunic biomass at LSR 50 (41.4 g/L), compared to 37.3 g/L for whole-tunic biomass [9]. Other marine biomass sources were studied for ethanol production such as aquatic plants or algae. The study carried out with aquatic plant *Eichhornia crassipes* has resulted in the ethanol concentration of 9.6 g/L [14]. For a different types of algae, *Laminaria japonica*, *Gracilaria* sp., and *Saccharina japonica,* the ethanol concentration was between 4.9 and 10.9 g/L [15–17]. Therefore, the results gained with organosolv pretreated tunicate biomass are 4–10 times higher.

### Production of prebiotic COS from outer tunic biomass

**Fig. 5:**
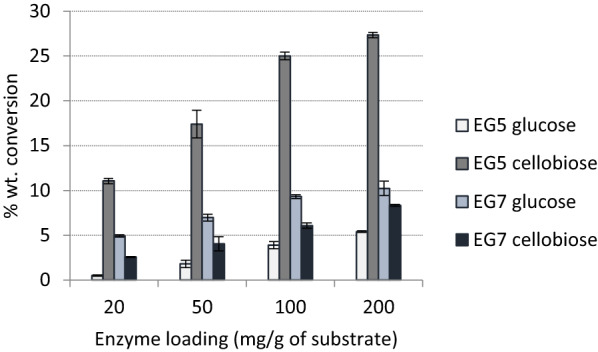
Mode of action of endoglucanase EG5 and EG7 on outer tunic. % wt. conversion of the substrate to glucose and cellobiose as a result of the effect of EG5 and EG7 action. The latter is a disaccharide with potential prebiotic activity

To evaluate the production of COS (mainly cellobiose; CB) with prebiotic potential from outer tunic biomass, first endoglucanases EG5 and EG7 were tested at different enzyme loadings (20, 50, 100, and 200 mg/g). After 24 h of hydrolysis, EG5 added at 20 mg/ g_solids_ led to release of 1.34 g/L cellobiose and 0.16 g/L glucose, corresponding to a conversion yield of 11.1 and 0.5%, respectively (Fig. 5). At an enzyme loading of 100 mg/ g_solids_, the amount of cellobiose was 2.88 g/L (25% cellulose conversion) with a ratio cellobiose to glucose (CB:Glc) equal to 6.4. On the contrary, EG7 showed a higher release of glucose than cellobiose in all reactions, reaching up to 10.2 and 8.3% substrate conversion in glucose and cellobiose, respectively. These results are in accordance with data from the literature describing the endo-action of GH7 endoglucanases that cleave glycosidic bonds that are located in the middle of the cellulose chain and perform one single cut per catalytic cycle [23]. On the contrary, GH5 endoglucanases with processive activity act on the same cellulose chain and release many soluble oligosaccharides before they detached from the substrate [24]. Therefore, EG5 was selected as a promising enzyme for cellobiose release and it was subsequently tested in combination with cellobiohydrolases CBHI and CBHII.

**Fig. 6:**
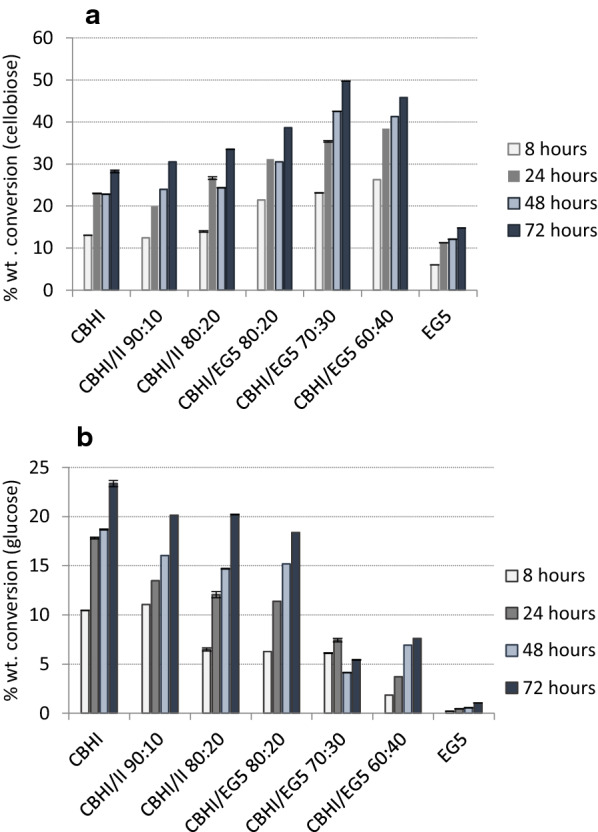
Effect of different enzyme combinations endoglucanase EG5 and cellobiohydrolases CBHI and CBHII on the conversion of outer tunic. Release of cellobiose (**a**) and glucose (**b**) from outer tunic by employing different enzyme combinations of endoglucanase EG5 and cellobiohydrolases CBHI and CBHII. The combination of CBHI/EG5 80:20 leads to maximal cellobiose release with minimum glucose yields

Different enzyme combinations were tested, while maintaining the total enzyme loading at 20 mg/g of substrate. It is observed that an addition of CBHII at 10% of the mixture brings a slight increase in cellobiose release (from 28.2 to 30.5% wt. at 72 h of hydrolysis), as depicted in Fig. 6. This effect is more profound when CBHII contribution rises up to 20%, leading to approx. 32% increase, which corresponds to a final yield of 3.7 g/L cellobiose and 33.5% wt. conversion. CB:Glc ratio when CBHI acts alone is close to 1.2 and increases upon addition of CBHII to 1.4. Synergistic effect of EG5 is higher than CBHII when these enzymes are added at 20% of the total enzyme loading. CBHI/EG5 80:20 results in 38.6% wt. conversion of cellulose to cellobiose after 72 h of hydrolysis with a ratio CB:Glc close to 2.1. The combination that reaches the maximal cellobiose yield is CBHI/EG5 80:20 with 49.7% wt. conversion and a CB:Glc ratio 9.4. These results indicate CBHI and EG5 as the two key enzymes for cellobiose production from outer tunic and they are in accordance with our previous results from forest biomass [19, 20]. It has been reported that hydrolysis with these enzymes led to 38% wt. cellobiose production from organosolv pretreated birch with a ratio of CBHI/EG5 60:40, and to 31% wt. cellobiose production from organosolv pretreated spruce with a ratio of CBHI/EG5 70:30 [20]. CBH is required in a higher relative amount for the conversion of tunicate compared to the forest biomass, most probably due to the high crystallinity of the tunicin, namely the cellulose isolated from tunicates [25]. CBH is the main enzyme that attacks the highly crystalline cellulose areas [26], which justifies its key role in the process. Set side by side, the production of cellobiose from tunicates has significant advantages over that from plant biomass, including, among others, the higher cellobiose yields and the recovery of a product free from lignin-derived phenolic compounds. The results of this study demonstrate the feasibility of obtaining cellobiose from tunicate. Moreover, the high yields of cellobiose render this substrate as a promising source of prebiotics and pave the way for the valorization of outer tunic for the production of value added products that can be used for food and feed supplements.

## Conclusion

Tunicate biomass possesses great potential as a feedstock for second-generation bioethanol production, as well as the isolation of cello-oligosaccharides with prebiotic potential. This study presents a convenient approach for improving ethanol concentrations and yield by fermenting organosolv-pretreated outer tunic biomass. The highest ethanol concentration, 41.4 g/L with yield of 90.2%, was obtained with outer tunic biomass pretreated at LSR 50. Regarding the production of COS, by employing a controlled enzymatic hydrolysis strategy with different combinations of cellulases as monoenzymes (endoglucanases and cellobiohydrolases), it was demonstrated that a maximum yield of 49.7 wt. % conversion of substrate to cellobiose is possible. Thus, the valorisation of tunicate biomass leads to an eco-friendly and sustainable process for production of value-added biofuels and chemicals.

## Material and methods

### Feedstock

Samples of *C. intestinalis* were collected East of the island of Tjörn on the Swedish West Coast in mid-autumn, October, (average salinity of 24–30 ppt). About 20 L of *C. intestinalis* specimens were boiled in 10 L of tap water for 4 min after reaching boiling point. Then, the water was poured out and the speciments were pressed between two rollers (diameter 140 mm), so the tissue body was pressed out of the tunic. During this pressing step, the mantle was also removed, resulting to successful recovery of the outer tunic. The resulting material was rinsed in fresh water for 4 min and then pressed in a nylon cloth (approx. 0.3-mm openings) using manual press force. Finally, the outer tunic was dried in a hot-air oven at 80 °C until moisture < 5% and ground to 3–75-mm flakes [9].

### Organosolv pretreatment

Organosolv pretreatment trials were performed with milled dried outer tunic biomass, which was treated at 195 °C in an air-heated multidigester system (AATO) with 6 × 2.5-L batch autoclave reactors at the Biochemical Process Engineering laboratories of the Chemical Engineering division of Luleå University of Technology. The pretreatment was carried out as described before [27]. Specifically treatment took place for 60 min with different liquid-to-solid ratios (LSRs; 10, 25, 50, 75) in 1.1 L of 60% v/v ethanol solution and varied amounts of tunic biomass to achieve the desired LSR. After the treatment time elapsed, the reactor was cooled down to below 40 °C. The pretreated biomass was separated from the liquor by vacuum filtration followed by a wash with 60% v/v ethanol and dried in the oven at 50 °C overnight. The dried samples were stored in plastic bottles. The ethanol from the filtrate was recovered by a rotary evaporator at 50 °C with pressure between 90 and 200 mbar (Heidolph, Schwabach, Germany). The solid precipitate was centrifuged from the aqueous solution at 15,000 × *g* for 10 min (Eppendorf centrifuge 5804R, Hamburk, Germany), freeze-dried, and stored at room temperature, while the remaining liquid was stored at 4 °C.

### Tunic compositional analysis

The untreated and pretreated tunicate biomass was analysed for cellulose content according to the protocol suggested by the National Renewable Energy Laboratory [28]. Carbohydrates were determined by HPLC (PerkinElmer, Waltham, USA) with a refractive index detector (PerkinElmer series 200,Waltham, USA) and an Aminex HPX-87H column (Bio-Rad, Hercules, CA, USA). The instrument was operated at 65 °C, with 5 mM H_2_SO_4_ as the mobile phase and a 0.6 mL/min flow rate. Total protein content was quantified according to the Kjeldahl method [29]. Lipids were determined gravimetrically [30]. The dry tunicate biomass was extracted for 4 h at room temperature with a solution of chloroform:methanol (1:2, v/v), followed by solvent evaporation in pre-weighted flasks. The inorganic ash was determined gravimetrically after treatment of the sample at 550 °C for 3 h in a furnace. The moisture was determined gravimetrically. The samples were placed in the oven at 80 °C overnight [9].

### Enzymatic saccharification for the production of fermentable sugars

Saccharification trials were performed to assess the potential of the pretreated solids in low-solid enzymatic saccharification. The trials were carried out in 2-mL Eppendorf tubes containing 1 mL of 3 wt. % dry solids in 50 mM citrate buffer (pH 5.0). The tubes were placed in a thermomixer (Eppendorf, Hamburg, Germany) for 48 h at 50 °C [9]. The commercial enzyme cocktail Cellic CTec2 (Novozyme A/S, Bagsværd, Denmark) was used at an enzyme loading of 20 FPU/g_solids_. The enzyme activity, as expressed in filter paper units (FPU), and the protein concentration of Cellic CTec2 were 238 FPU/mL and 278 mg/mL, respectively [27]. The sugar profile was analysed every 24 h of incubation by high-performance liquid chromatography (HPLC) (as described above).

In order to test the highest hydrolysability of the outer tunic, reactions with 1 wt. % dry solids were designed by using the outer tunic treated at LSR 75. Cellobiohydrolase I (CBH7) from *Trichoderma longibrachiatum* was purchased from Megazyme (Bray, Ireland). Total enzyme loading of Cellic CTec2 and CBHI was 20 mg/g_solids_ in all different combinations. Reactions took place at 50 °C, 1100 rpm, in 100 mM phosphate-citrate buffer pH 5.0 [24]. Samples were taken at 4, 8 and 24 h, centrifuged at 8000 × *g* for 10 min (Eppendorf centrifuge 5804R, Hamburk, Germany). The supernatant was analyzed for glucose content with HPLC as described above.

For the production of ethanol, saccharification reaction was scaled up prior to the fermentations trials [9]. Enzymatic saccharification was performed with Cellic CTec2, in 50-mL Erlenmeyer flasks containing 20 g of 15 wt.% dry solids in 50 mM citrate buffer (pH 5.0) for 24 h at 50 °C with an enzyme loading of 20 FPU/g_solids_.

### Ethanol fermentation

The yeast strain *Saccharomyces cerevisiae* Ethanol Red (purchased from Lesaffre, Marcq-en-Barœul, France) was used for the fermentation trials [9]. The strain was stored at 4 °C as a lyophilized pellet. Prior to inoculation, the yeast was grown on yeast extract-peptone-dextrose (YPD) medium at 35 °C for 24 h. Subsequently, the cells were harvested by centrifugation (8000 × *g*, 10 min, 20 °C) and inoculated (1 g_DCW_/L) in the cooled saccharification slurry (35 °C). Then, the slurry was supplemented with MgSO_4_‧7H_2_O (0.025 g/L), (NH_4_)_2_HPO_4_ (0.5 g/L), and yeast extract (1 g/L) and placed in an orbital shaker (Zhicheng, Shanghai, China) at 35 °C and 200 rpm. Samples were taken every 24 h and analysed for ethanol and residual sugars by HPLC (described above). The maximal theoretical yield was calculated by taking into account the stoichiometric conversion of glucose to ethanol (yielding a maximum of 0.511 g/g). Thus, the ethanol yields are the comparison of the measured ethanol concentrations to the maximal theoretical ethanol concentration.

### Fine-tuned enzymatic production of cello-oligosaccharides

All reactions took place at 1 wt. % dry solids. Two endoglucanase (EG5, EG7) belonging to GH5 and seven family from the filamentous fungi *Thermothelomyces thermophila*, heterologously expressed in *Pichia pastoris,* were used in different enzyme loadings, varying from 20 to 200 mg/g_solids_ [24, 31]. Cellobiohydrolase I (CBH7) from *Trichoderma longibrachiatum* and recombinant cellobiohydrolase II (CBH6) were purchased from Megazyme (Bray, Ireland). Reactions with different enzyme combinations occurred at a total enzyme loading of 20 mg/g_solids_. Reactions took place at 50 °C, 1100 rpm, in 100 mM phosphate-citrate buffer pH 5.0 [24]. Samples were taken at 24 h, centrifuged at 8000 × *g* for 10 min (Eppendorf centrifuge 5804R, Hamburk, Germany). The supernatant was analyzed for cellobiose and glucose content with HPLC as described above.

## Data Availability

All data supporting the conclusions of this article are included in the manuscript. Samples of materials produced in the current work are available from the corresponding author upon reasonable request.

## References

[1] Kohli K, Prajapati R, Sharma BK (2019). Bio-based chemicals from renewable biomass for integrated biorefineries. Energies.

[2] Ragauskas AJ, Williams CK, Davison BH, Britovsek G, Cairney J, Eckert CA (2006). The path forward for biofuels and biomaterials. Science.

[3] United Nations. Energy—United Nations Sustainable Development. UN. 2017. https://www.un.org/sustainabledevelopment/energy/.

[4] Cellulosic Ethanol - Iogen Corporation. Technology Scale-up and Validation. https://iogen.ca/cellulosic_ethanol/scale-up.html. Accessed 20 January 2021.

[5] Kalundborg Bioethanol Demonstration Plant. https://www.chemicals-technology.com/projects/kalundborg_bioethano/. Accessed 20 January 2021.

[6] DuPont closes Iowa cellulosic ethanol plant. https://www.iowafarmbureau.com/Article/DuPont-closes-Iowa-cellulosic-ethanol-plant. Accessed 20 January 2021.

[7] Binod P, Gnansounou E, Sindhu R, Pandey A (2019). Enzymes for second generation biofuels: recent developments and future perspectives. Bioresour Technol Rep.

[8] Leong WH, Lim JW, Lam MK, Uemura Y, Ho YC (2018). Third generation biofuels: a nutritional perspective in enhancing microbial lipid production. Renew Sustain Energy Rev.

[9] Hrůzová K, Matsakas L, Karnaouri A, Norén F, Rova U, Christakopoulos P (2020). Second-generation biofuel production from the marine filter feeder Ciona intestinalis. ACS Sustain Chem Eng.

[10] Troedsson C, Thompson E, Bouquet JM, Magnesen T, Schander C, Li J. Tunicate extract for use in animal feeds. 2013.

[11] Marine Feed. https://marinefeed.com/. Accessed 20 January 2021.

[12] Marin Biogas. http://www.marinbiogas.se/en

[13] Zhao Y, Li J (2016). Ascidian bioresources: common and variant chemical compositions and exploitation strategy—examples of *Halocynthia roretzi*, *Styela plicata*, *Ascidia* sp. and *Ciona intestinalis*. Zeitschrift fur Naturforsch..

[14] Takagi T, Uchida M, Matsushima R, Ishida M, Urano N (2012). Efficient bioethanol production from water hyacinth *Eichhornia crassipes* by both preparation of the saccharified solution and selection of fermenting yeasts. Fish Sci.

[15] Lee SM, Lee JH (2012). Ethanol fermentation for main sugar components of brown-algae using various yeasts. J Ind Eng Chem.

[16] Wu FC, Wu JY, Liao YJ, Wang MY, Shih IL (2014). Sequential acid and enzymatic hydrolysis in situ and bioethanol production from Gracilaria biomass. Bioresour Technol.

[17] Lee J, Li P, Lee J, Ryu HJ, Oh KK (2013). Ethanol production from Saccharina japonica using an optimized extremely low acid pretreatment followed by simultaneous saccharification and fermentation. Bioresour Technol..

[18] Swennen K, Courtin CM, Delcour JA (2006). Non-digestible oligosaccharides with prebiotic properties. Crit Rev Food Sci Nutr.

[19] Karnaouri A, Matsakas L, Krikigianni E, Rova U, Christakopoulos P (2019). Valorization of waste forest biomass toward the production of cello-oligosaccharides with potential prebiotic activity by utilizing customized enzyme cocktails. Biotechnol Biofuels.

[20] Karnaouri A, Topakas E, Matsakas L, Rova U, Christakopoulos P (2018). Fine-tuned enzymatic hydrolysis of organosolv pretreated forest materials for the efficient production of cellobiose. Front Chem.

[21] Hörhammer H, Dou C, Gustafson R, Suko A, Bura R (2018). Removal of non-structural components from poplar whole-tree chips to enhance hydrolysis and fermentation performance. Biotechnol Biofuels.

[22] Zhang YHP, Lynd LR (2004). Toward an aggregated understanding of enzymatic hydrolysis of cellulose: noncomplexed cellulase systems. Biotechnol Bioeng.

[23] Bu L, Nimlos MR, Shirts MR, Ståhlberg J, Himmel ME, Crowley MF (2012). Product binding varies dramatically between processive and nonprocessive cellulase enzymes. J Biol Chem.

[24] Karnaouri A, Muraleedharan MN, Dimarogona M, Topakas E, Rova U, Sandgren M (2017). Recombinant expression of thermostable processive MtEG5 endoglucanase and its synergism with MtLPMO from *Myceliophthora thermophila* during the hydrolysis of lignocellulosic substrates. Biotechnol Biofuels.

[25] Zhao Y, Li J (2014). Excellent chemical and material cellulose from tunicates: diversity in cellulose production yield and chemical and morphological structures from different tunicate species. Cellulose.

[26] Sinnott ML (1998). The cellobiohydrolases of *Trichoderma reesei*: a review of indirect and direct evidence that their function is not just glycosidic bond hydrolysis. Biochem Soc Trans..

[27] Matsakas L, Nitsos C, Vörös D, Rova U, Christakopoulos P (2017). High-titer methane from organosolv-pretreated spruce and birch. Energies.

[28] Sluiter A, Hames B, Ruiz R, Scarlata C, Sluiter J, Templeton D, et al. Determination of Structural Carbohydrates and Lignin in Biomass: Laboratory Analytical Procedure (LAP). 2008.

[29] Kjeldahl J (1883). Neue Methode zur Bestimmung des Stickstoffs in organischen Körpern. Zeitschrift für Anal Chemie.

[30] Patel A, Hrůzová K, Rova U, Christakopoulos P, Matsakas L (2019). Sustainable biorefinery concept for biofuel production through holistic volarization of food waste. Bioresour Technol.

[31] Karnaouri AC, Topakas E, Christakopoulos P (2014). Cloning, expression, and characterization of a thermostable GH7 endoglucanase from *Myceliophthora thermophila* capable of high-consistency enzymatic liquefaction. Appl Microbiol Biotechnol.

